# Persistent executive, visuospatial, and conceptual deficits after right posterior cerebellar infarction

**DOI:** 10.1002/pcn5.70381

**Published:** 2026-07-16

**Authors:** Daisuke Yoshioka, Yuji Higaki, Masaaki Iwata

**Affiliations:** ^1^ Department of Psychiatry Yowa Hospital Tottori Japan; ^2^ Department of Neuropsychiatry, Faculty of Medicine Tottori University Tottori Japan; ^3^ Department of Neurology Yowa Hospital Tottori Japan

**Keywords:** cerebellar cognitive affective syndrome, cerebellar infarction, cognitive dysfunction, conceptual deficits, posterior inferior cerebellar artery

## Abstract

**Background:**

The cerebellum has traditionally been regarded as a structure primarily involved in motor coordination. However, accumulating evidence indicates that the cerebellum can exert substantial influence on cognitive and emotional processes. These findings suggest that the cerebellum may play a broader role in higher‐order cognitive functions than previously recognized.

**Case Presentation:**

A right‐handed Japanese man in his 80s with no significant prior medical history developed cognitive and behavioral symptoms following an infarction involving the right posterior cerebellar lobe and vermis, corresponding to the right posterior inferior cerebellar artery territory. Emotional lability, disorientation, and memory impairment emerged during the subacute phase but gradually improved. In contrast, executive dysfunction, perseveration, and visuospatial impairment persisted. On the Clock Drawing Test, errors such as misplacement of numbers and incorrect representation of time were observed, which could not be explained by visuospatial deficits and suggested impairment of conceptual aspects of cognition.

**Conclusion:**

This case demonstrates that a right posterior cerebellar infarction can result in persistent impairments in executive, visuospatial, and conceptual functions. These findings suggest that cerebellar injury may affect higher‐order cognition not only through disruption of cerebello‐cerebral connectivity, but also through impairment of the cerebellum's modulatory role in the regulation of complex cognitive operations.

## BACKGROUND

Historically, the cerebellum was considered responsible mainly for motor coordination, balance, and gait. As evidence accumulated that cerebellar damage can cause cognitive and emotional disturbances, Schmahmann and Sherman proposed cerebellar cognitive affective syndrome (CCAS).^1^ This syndrome is characterized by impairments in executive function, visuospatial ability, language, and emotion. Subsequent neuroanatomical and neuroimaging studies have demonstrated extensive reciprocal connections between the cerebellum and association cortices, providing an anatomical substrate for higher‐order cerebellar functions.^2^ Although CCAS has been reported for over two decades, the precise contribution of the cerebellum to higher‐order cognitive functions remains incompletely understood.

We report a case of an elderly man who developed cognitive disturbances after a right posterior inferior cerebellar artery (PICA) infarction. This case illustrates diverse neuropsychiatric manifestations of cerebellar lesions and provides insight into cerebellar contributions to cognition.

## CASE PRESENTATION

A right‐handed Japanese man in his 80s with no significant medical history had been living independently and working daily on his family farm. He managed finances independently, and his family had noticed no signs of cognitive decline prior to hospitalization. Two months before admission, he developed bilateral leg edema but declined medical evaluation. When his family later found him at home with dyspnea and malaise, they brought him to the hospital. He was diagnosed with an acute exacerbation of chronic heart failure due to an old myocardial infarction and was admitted to the cardiology ward.

Several days after admission, he developed truncal and right‐sided limb ataxia. Brain magnetic resonance imaging (MRI) showed a recent infarction involving the right posterior cerebellar lobe and vermis, corresponding to the right PICA territory, with occlusion of the right vertebral artery. Hippocampal atrophy was also noted (Figure 1).

**Figure 1:**
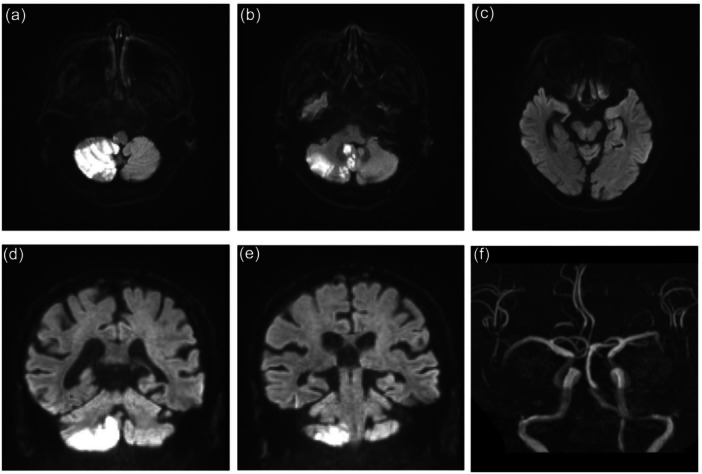
Diffusion‐weighted magnetic resonance imaging and magnetic resonance angiography findings. (a–e) Diffusion‐weighted images demonstrate a hyperintense lesion in the right posterior cerebellar hemisphere involving the inferior vermis, consistent with an acute infarction in the posterior inferior cerebellar artery (PICA) territory. Hippocampal atrophy is also noted. (f) Magnetic resonance angiography shows non‐visualization of the right vertebral artery. All images were acquired using a 3.0‐T MRI scanner (MAGNETOM Skyra, Siemens Healthineers). Diffusion‐weighted imaging was performed with axial 5 mm and coronal 3 mm slices, three orthogonal diffusion‐encoding directions, and a b‐value of 1000 s/mm^2^.

Intravenous argatroban therapy was started. Six weeks later, he fell. Head computed tomography (CT) the next day revealed a small left acute subdural hematoma (Supporting Information S1: Figure S1), which was managed conservatively. Subsequently, he developed emotional lability and agitation. Psychiatrists initiated blonanserin (20 mg patch), quetiapine (25 mg), and valproate (400 mg), but these behavioral symptoms persisted. Consequently, he was transferred to our psychiatric department approximately ten weeks after the initial hospitalization for further management and care planning.

Three days after transfer, his mental state stabilized, and he was calm, cooperative, and fully alert. Follow‐up CT showed the known right cerebellar infarction and no substantial change in the left subdural hematoma (Supporting Information S1: Figure S1). Speech was slightly dysfluent but without scanning or explosive characteristics. Neurological examination revealed moderate right‐sided limb ataxia on finger‐to‐nose and heel‐to‐shin testing. Tandem gait was difficult, and Mann's test was positive. Arm swing was slightly reduced on the right. Neuropsychological testing one week after admission revealed marked cognitive impairment. The mini‐mental state examination (MMSE) score was 13/30, showing severe deficits across multiple cognitive domains. Perseveration was evident on the clock drawing test (CDT): immediately after copying a pentagon in the MMSE, he drew multiple overlapping pentagons instead of a clock. The frontal assessment battery (FAB) score was 7/18, indicating impairment across multiple frontal executive domains, except for prehension behavior.

Based on the clinical course, neuropsychological findings, and neuroimaging results, we diagnosed the patient with CCAS. The patient developed emotional lability and agitation after the acute subdural hematoma. These symptoms were transient and resolved shortly after transfer to our psychiatric department. We considered them to reflect a transient confusional state related to the subdural hematoma. In contrast, we considered the persistent cognitive impairment observed after transfer unlikely to be explained solely by the subdural hematoma. The hematoma remained small, caused minimal mass effect, and showed no progression on serial neuroimaging. Although we could not completely exclude the possibility of delirium given the patient's age and concomitant medical illnesses, he remained fully alert, calm, and cooperative throughout his hospitalization, and we observed no marked fluctuation in his mental status. Therefore, we considered delirium unlikely to explain the persistent cognitive impairment. Neuroimaging showed hippocampal atrophy. However, neither the patient nor his family had noticed any cognitive decline before the onset of the cerebellar infarction, and he had remained fully independent in daily life. Therefore, although we could not completely exclude a contribution of hippocampal atrophy to the cognitive symptoms, we considered its impact limited.

One month later, his MMSE improved to 19/30, mainly reflecting better orientation and immediate registration, while deficits in other cognitive domains persisted. Executive dysfunction remained severely impaired (FAB 5/18). Perseveration remained. In the MMSE writing task, instead of generating a spontaneous sentence, he wrote “Close eyes,” repeating the immediately preceding written command (“Close your eyes”). On the CDT, he failed to draw an accurate clock face or hands; the numbers were sparsely placed outside the clock outline, and no clock hands were drawn. Instead, he wrote the number “10” beneath “11,” explaining that this was intended to represent the time “11:10.” This finding suggested an impaired understanding of the analog clock concept, in which time is represented by numbers and clock hands. Two months after admission (6 months after onset of the infarction), motor symptoms had almost completely resolved; gait was stable and right‐hand clumsiness improved. Because residual cognitive impairment persisted, he was discharged to a long‐term care facility. At discharge, the MMSE score was 22/30, reflecting improvement in delayed recall. The same perseverative response was again observed on the writing task, with the patient reproducing the immediately preceding written command (“Close your eyes”). On the CDT, a similar error pattern was observed, with the numbers written outside the clock outline and no clock hands drawn. He drew a dot‐like mark next to the number “11” and explained that it was intended to represent “11:10.” Overall, the neuropsychological profile demonstrated persistent deficits in executive function, visuospatial ability, and conceptual processing. Detailed results of the MMSE and FAB are presented in Table 1, and characteristic findings from the neuropsychological assessments are shown in Figure 2.

**Table 1:** Serial changes in MMSE and FAB scores.

	1 week after admission	1 month after admission	2 months after admission
MMSE (total score)/30	13	19	22
Date orientation/5	2	4	5
Place orientation/5	3	4	4
Registration 3 objects/3	1	3	3
Serial 7/5	1	1	1
Recall 3 objects/3	0	0	2
Naming/2	1	2	2
Repetition/1	1	1	1
Verbal commands/3	3	3	3
Written commands/1	1	1	1
Writing/1	0	0	0
Pentagon copying/1	0	0	0
FAB (total score)/18	7	5	–
Similarities/3	1	1	–
Lexical fluency/3	0	0	–
Motor series/3	1	1	–
Conflicting instructions/3	1	0	–
Go no Go/3	1	0	–
Prehension behavior/3	3	3	–

*Note*: Values represent raw subscores for each cognitive domain. – indicates that the FAB was not assessed at this time point.

Abbreviations: FAB, frontal assessment battery; MMSE, mini‐mental state examination.

**Figure 2:**
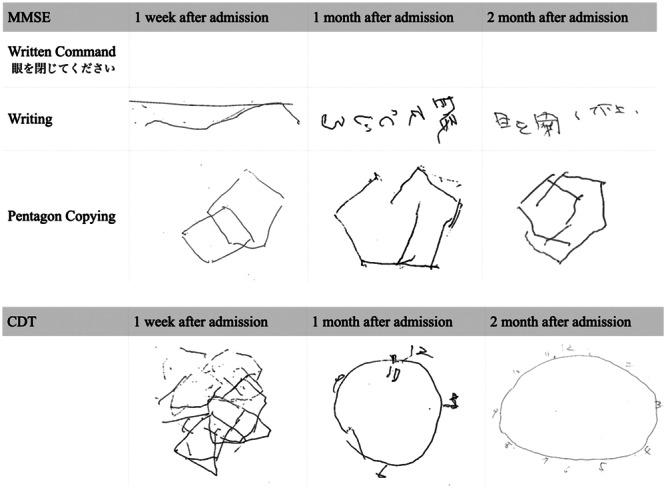
Serial changes in MMSE writing and pentagon copying, and in the CDT. This figure shows serial changes in the patient's performance on MMSE writing and pentagon copying tasks and on the CDT at 1 week, 1 month, and 2 months after admission. The written command in the MMSE was “Close your eyes” (Japanese). MMSE tasks: 1 week after admission: The patient was unable to produce a meaningful sentence in the writing task. In the Pentagon copying task, inaccuracy was observed, and the figure could not be reproduced correctly. 1 month after admission: In the writing task, which required the patient to generate a spontaneous sentence, he wrote “Close eyes.” This represented perseveration of the immediately preceding written command (“Close your eyes”). The pentagon copying task remained inaccurate. Two months after admission: In the writing task, the patient again wrote “Close your eyes,” indicating persistent perseveration. The copied pentagon also remained inaccurate. CDT tasks: 1 week after admission: The drawing included multiple overlapping pentagon‐like figures, reflecting perseveration of the preceding pentagon copying task rather than an attempt to draw a clock. One month after admission: The patient drew an approximately circular outline, but the numbers were sparsely placed outside the clock outline, and no clock hands were drawn. Although the numerals were distorted and difficult to read, he wrote the number “10” beneath “11” and explained that this was intended to indicate the time “11:10.” Two months after admission: A clock outline was present, but all numbers were written outside the clock outline, and no clock hands were drawn. A small black dot was placed next to the number “11”; when asked about its meaning, the patient explained that it was intended to represent “10,” reflecting an attempt to depict the time “11:10.” CDT, clock drawing test; MMSE, mini‐mental state examination.

## DISCUSSION

This elderly patient developed significant cognitive impairment following an infarction in the right PICA territory. Although orientation and memory functions showed substantial improvement over time, executive dysfunction, visuospatial impairment, and conceptual deficits persisted 6 months after onset.

Conceptual impairment emerged as a prominent feature in this case. The patient's CDT drawings demonstrated impaired understanding of the fundamental meaning of a clock, highlighting a potentially underrecognized aspect of CCAS.

Neuroimaging and lesion studies have shown that cognitive impairment caused by cerebellar damage is primarily associated with posterior cerebellar lesions, whereas anterior lobe damage mainly produces motor symptoms.^3^ Moreover, infarctions in the PICA territory have been reported to cause more pronounced cognitive and emotional disturbances than those in the superior cerebellar artery territory, suggesting that the posterior cerebellum plays a key role in higher cognitive and emotional processing.^4^ The severe cognitive impairment observed in this case is consistent with these previous findings.

Memory impairment has been reported in cerebellar lesions but may reflect deficits in attention or executive function rather than primary memory dysfunction,^5^ and impairment of semantic and episodic memory is considered atypical in cerebellar disorders.^6^ Because MMSE memory tasks require sustained attention, interpretation of primary memory impairment should be cautious. Involvement of cerebello‐cerebral networks, including reciprocal pathways with the hippocampus^2^, ^7^ and dysfunction of the thalamus as a relay center,^8^ has been proposed.

Neuropsychological testing revealed impairment of frontal lobe function. Disruption of the circuits connecting the cerebellum and prefrontal cortex has been reported to impair planning, attentional shifting, and inhibitory control, leading to executive dysfunction resembling that observed in frontal lobe damage.^1^, ^9^ The right posterior cerebellum is thought to contribute to executive control and the regulation of working memory.^10^, ^11^, ^12^ In the present case, impairment of lexical fluency was observed on the FAB. Although lexical fluency is categorized as an executive function, it also reflects language processing. Given accumulating evidence that the cerebellum plays a modulatory role in language processing,^13^ the reduced lexical fluency observed in this case may reflect cerebellar involvement in language‐related cognitive functions, in addition to executive dysfunction. Furthermore, the patient exhibited perseverative behavior characterized by repetitive and inappropriate responses during the MMSE and CDT. These findings can be interpreted using the “dysmetria of thought” hypothesis.^14^ This concept proposes that the cerebellum regulates the precision and timing of cognitive operations, analogous to its role in motor coordination. Moreover, the cerebellum has been implicated in the generation and selection of “candidate responses” during memory retrieval, and damage to this system may result in distorted or repetitive outputs.^15^ These findings suggest that perseverative behavior reflects impaired cognitive control due to cerebellar dysfunction.

Visuospatial impairment was another prominent feature. Previous studies have shown that cerebellar lesions, particularly in the posterior lobe, can impair tasks involving spatial organization, mental rotation, and constructional ability while sparing basic visual perception.^16^

On the CDT, the patient exhibited errors that could not be explained by visuospatial impairment alone, including placing numbers outside the clock outline, omission of clock hands, and attempting to represent the time in a digital manner. These findings suggest a conceptual deficit reflecting impaired access to the attributes, features, and meaning of an analog clock. Rouleau et al. described such “misrepresentation of time” as a loss of semantic knowledge regarding the fundamental function of a clock—to communicate time.^17^ Conceptual errors on the CDT have been associated with disturbances in semantic knowledge^18^ and are frequently observed in Alzheimer's disease.^17^ Conceptual impairment has rarely been emphasized as a core feature of cognitive dysfunction following cerebellar damage. In the present case, the use of the patient's original CDT drawings enabled direct visualization of a disturbance in conceptual knowledge rather than a mere failure of visuospatial construction, thereby highlighting conceptual impairment as a potentially underrecognized component of cerebellar cognitive dysfunction.

Beyond these domain‐specific findings, the pattern of cognitive impairment observed in the present case raises important questions regarding the mechanisms underlying diverse and domain‐specific cognitive deficits following cerebellar damage. Cognitive impairment associated with cerebellar lesions has often been discussed from the perspective of disrupted connectivity between the cerebellum and the cerebral cortex. However, the overall clinical course suggests that cerebello–cortical disconnection alone cannot fully account for the observed pattern of recovery. While executive dysfunction with prominent perseveration, visuospatial impairment, and conceptual errors persisted, other cognitive domains such as orientation and memory showed improvement over time. If these deficits were attributable solely to stable disconnection from specific cortical regions, a more uniform persistence of cognitive impairment might be expected. One plausible interpretation is that the cerebellum contributes to the precise modulation and context‐appropriate execution of cognitive operations. From this perspective, relatively simple cognitive functions such as orientation and basic memory registration may be more readily compensated by cerebral cortical systems. In contrast, complex executive functions, higher‐order visuospatial processing, and conceptual operations may depend more strongly on cerebellar modulation. Disruption of this modulatory role may therefore selectively impair complex cognitive operations that are difficult to compensate for, helping to explain the persistence of executive, visuospatial, and conceptual deficits in this case despite recovery in other cognitive domains following posterior cerebellar infarction.

A limitation of this report is that it describes a single case, and the generalizability of the findings is therefore limited. In addition, a standardized assessment using the CCAS/Schmahmann syndrome scale was not performed. Although the CCAS scale is a useful tool for detecting cognitive impairment related to cerebellar dysfunction,^19^ it was not used because a Japanese version of the scale has not yet been published. Finally, long‐term longitudinal assessment could not be conducted because the patient was discharged and transferred to a long‐term care facility.

## CONCLUSION

This case illustrates that a right posterior cerebellar infarction can lead to persistent cognitive impairments, including executive dysfunction, visuospatial impairment, and conceptual disturbance. These findings suggest that the cerebellum contributes to higher‐order cognitive functions not only through its interactions with cerebral association networks, but also through its modulatory role in the regulation of complex cognitive operations.

## AUTHOR CONTRIBUTIONS

Daisuke Yoshioka and Yuji Higaki treated the patient. Daisuke Yoshioka drafted the manuscript. Yuji Higaki and Masaaki Iwata critically reviewed the draft and revised it.

## CONFLICT OF INTEREST STATEMENT

The authors declare no conflicts of interest.

## ETHICS APPROVAL STATEMENT

This case report was conducted in accordance with the principles of the Declaration of Helsinki and its later amendments. Formal ethics committee approval was not required for this single‐patient case report, as it was conducted as part of routine clinical care, in accordance with institutional policy.

## PATIENT CONSENT STATEMENT

Informed written consent and a signed release were obtained from the patient's family for the publication of this report and any accompanying images.

## CLINICAL TRIAL REGISTRATION

N/A.

## Supporting information

Supplementary Information.

## Data Availability

Data sharing is not applicable to this article as no datasets were generated or analyzed during the current study. No datasets were generated or analyzed for this case report.
