# Supplementary material for: Persistent executive, visuospatial, and conceptual deficits after right posterior cerebellar infarction
Source: PCN Rep. 2026 Jul 16;5(3):e70381. doi: 10.1002/pcn5.70381 (PMC13373931; doi:10.1002/pcn5.70381)
Supplement: Supplementary file 1 — Supplementary Information. [file PCN5-5-e70381-s001.docx]

**Supplementary Figure 1
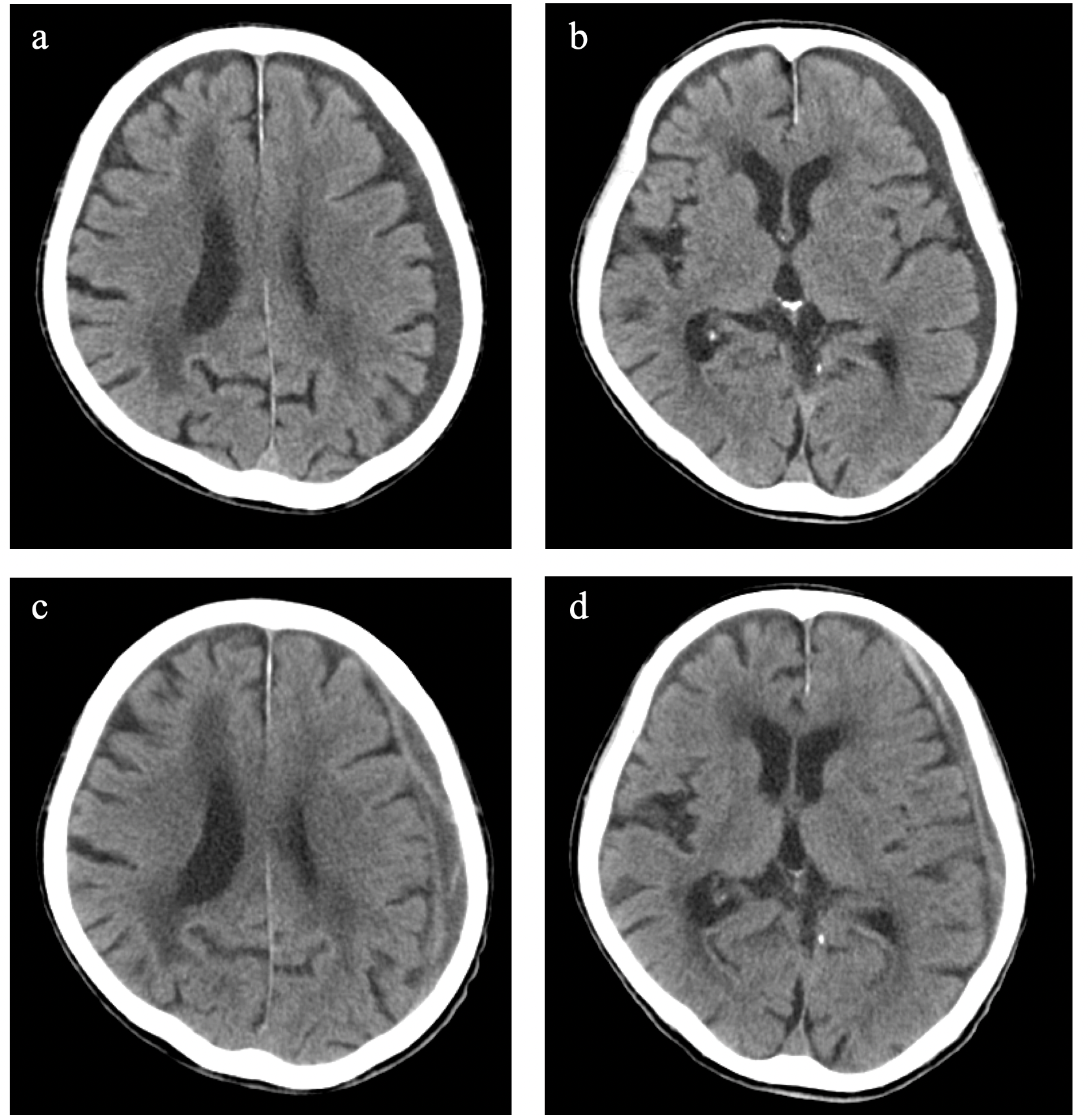
: Serial CT findings of the subdural hematoma**

(a, b) Computed tomography (CT) of the head performed after the fall shows a small left-sided acute subdural hematoma with minimal midline shift and no significant mass effect.

(c, d) Follow-up CT obtained approximately 4 weeks after the initial detection of the hematoma, following admission to our hospital, demonstrates no interval enlargement of the hematoma and no progression of mass effect or midline shift compared with the previous examination.
